# Cryoglobulinemia, monoclonal and mixed cryoglobulinemia syndromes, cryoglobulinemic vasculitis: *a proposal for comprehensive nomenclature and definition*

**DOI:** 10.3389/fimmu.2026.1754012

**Published:** 2026-02-20

**Authors:** Clodoveo Ferri, Laura Gragnani, Anna Linda Zignego, Dilia Giuggioli

**Affiliations:** 1 Rheumatology Unit, Policlinico di Modena, University of Modena & Reggio E., Modena, Italy; 2 Rheumatology Unit, Casa di Cura Madonna dello Scoglio, Crotone, Italy; 3 Department of Translational Research and New Medical and Surgical Technologies, University of Pisa, Pisa, Italy; 4 Department of Experimental and Clinical Medicine, University of Florence, Florence, Italy

**Keywords:** cryoglobulinemia, cryoglobulinemic vasculitis, mixed cryoglobulinaemia, monoclonal cryoglobulinemia, nomenclature

## Abstract

**Background:**

Cryoglobulinemia indicates the reversible, cold-dependent precipitation of immunoglobulins (Ig), which may be monoclonal (MoC) or mixed IgG–IgM (MC). Although this *in vitro* phenomenon is relatively frequent, only a minority of cases develop clinically relevant manifestations, often falling within lymphoproliferative, autoimmune, or infectious disease domains. This complexity highlights the need for clearer nomenclature and definition systems.

**Need for Standardized Nomenclature and Definitions:**

The term “cryoglobulinemia” is often used interchangeably to describe either simple laboratory finding, asymptomatic cryo-Ig precipitation (MoC or MC) or corresponding clinical syndromes (MoCs or MCs). This overlap creates ambiguity, hinders expert communication, and complicates the comparison of clinical studies. A standardized nomenclature is therefore essential to define disease subsets, establish boundaries with related conditions, and enable reliable data collection and multicenter analyses.

**Assessment of Cryoglobulinemia:**

When cryoglobulins are detected, the cryoprecipitate must be isolated and defined as MoC or MC, followed by a systematic clinical and laboratory assessment to identify potential underlying disorders. Immunoserological, microbiological, and pathological investigations are required to classify both MoC and MC as asymptomatic or symptomatic, and as essential or secondary. Asymptomatic individuals must be monitored over time for potential progression to overt clinical syndromes; similarly, those with essential forms require surveillance for the emergence of systemic diseases. Possible overlap with autoimmune conditions, particularly Sjögren’s disease, should always be considered.

**Two Distinct Disorders:**

Despite some shared features, MoCs and MCs represent distinct clinical entities. Accurate diagnosis relies primarily on cryoprecipitate analysis. MoCs are usually associated with thrombotic, non-inflammatory microangiopathy and lymphoproliferative disorders, whereas MCs are characterized by immune-complex–mediated leukocytoclastic vasculitis, complement consumption, and frequent association with viral infections, especially HCV and HBV. The introduction of new-generation antivirals in the last decade has markedly reduced the prevalence of virus-related MCs in more developed countries.

**Conclusions:**

A harmonized nomenclature and standardized definitions for various cryoglobulinemia subsets, supported by careful clinical-immunological and pathological characterization, are essential to improve diagnostic accuracy, ensure interobserver consistency, and enable the development of evidence-based, mechanism-driven therapies for major cryoglobulinemic syndromes.

## 1 Introduction

**Figure 1:**
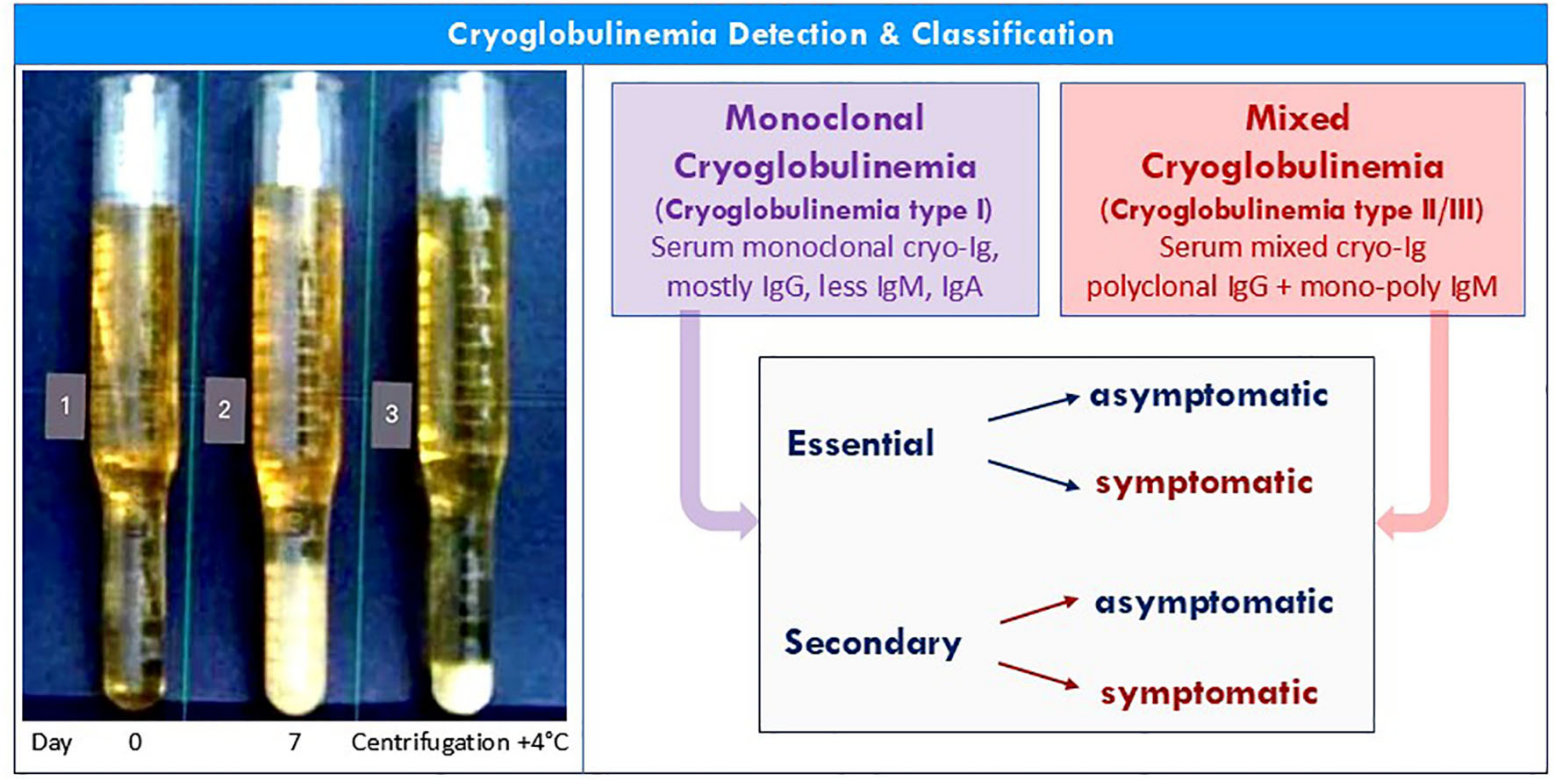
Detection & classification of cryoglobulinemia. Left: given their high thermal instability, a correct detection and characterization of circulating cryoglobulins must adopt some important precautions (see text). 1. Isolated serum (blood sampling, clotting and serum separation by centrifugation should always be carried out at 37 °C to avoid false-negative results); 2. Cryoprecipitate after 7 days storage at 4 °C; 3. cryocrit determination after centrifugation (percentage of packed cryoglobulins in serum sample stored at 4 °C for 7 days; cryocrit should be determined on blood samples without anticoagulation to avoid false-positive results due to cryofibrinogen or heparin-precipitable proteins). Right. The cryoglobulinemia is primarily classified according to the immunoglobulin composition as monoclonal, type I, cryoglobulinemia or mixed, type II/III, cryoglobulinemia, which should be regarded as two distinct immunological disorders. These distinct serotypes should be classified as ‘essential’ or ‘secondary’ in the absence/presence of well-known underlying disorders (B-cell lymphoid neoplasms, infectious diseases, and/or systemic autoimmune disorder), which in turn are classified as ‘‘symptomatic’ or ‘asymptomatic’ according to the presence/absence of one or more typical clinical/pathological manifestations (see also **Figure 2** and text).

The presence in the serum of one or more immunoglobulins that precipitate at temperatures below 37 °C and redissolve on rewarming is termed cryoglobulinemia (**Figure 1**) (1–6)(Zignego AL et al. J. Int. Medicine 2025, accepted for publication). The actual mechanism of this *in vitro* phenomenon, first described by Wintrobe and Buell in 1933 (7), remains quite obscure; the immunoglobulin (Ig) cryoprecipitation could be secondary to intrinsic characteristics of both monoclonal and polyclonal Ig and/or caused by the interaction among different components of the cryoprecipitate. The most accredited explanations of this phenomenon, not mutually exclusive, include the following pathogenetic mechanisms: structural modification of the variable portions of Ig heavy/light chains, reduced amounts of galactose in the Fc portion of the Ig, reduced concentration of sialic acid, and/or the presence of N-linked glycosylation sites in the CH3 domain secondary to somatic Ig mutations during autoimmune responses (1–7). The Ig self-aggregation might also be explained by both non-specific Fc–Fc interactions, or as for mixed IgG-IgM cryoglobulinemia (MC), by specific interactions among IgM rheumatoid factor (RF) and Fc portion of IgG, respectively the autoantibody and the autoantigen of cryoprecipitable immune-complexes (1–7).

**Figure 2:**
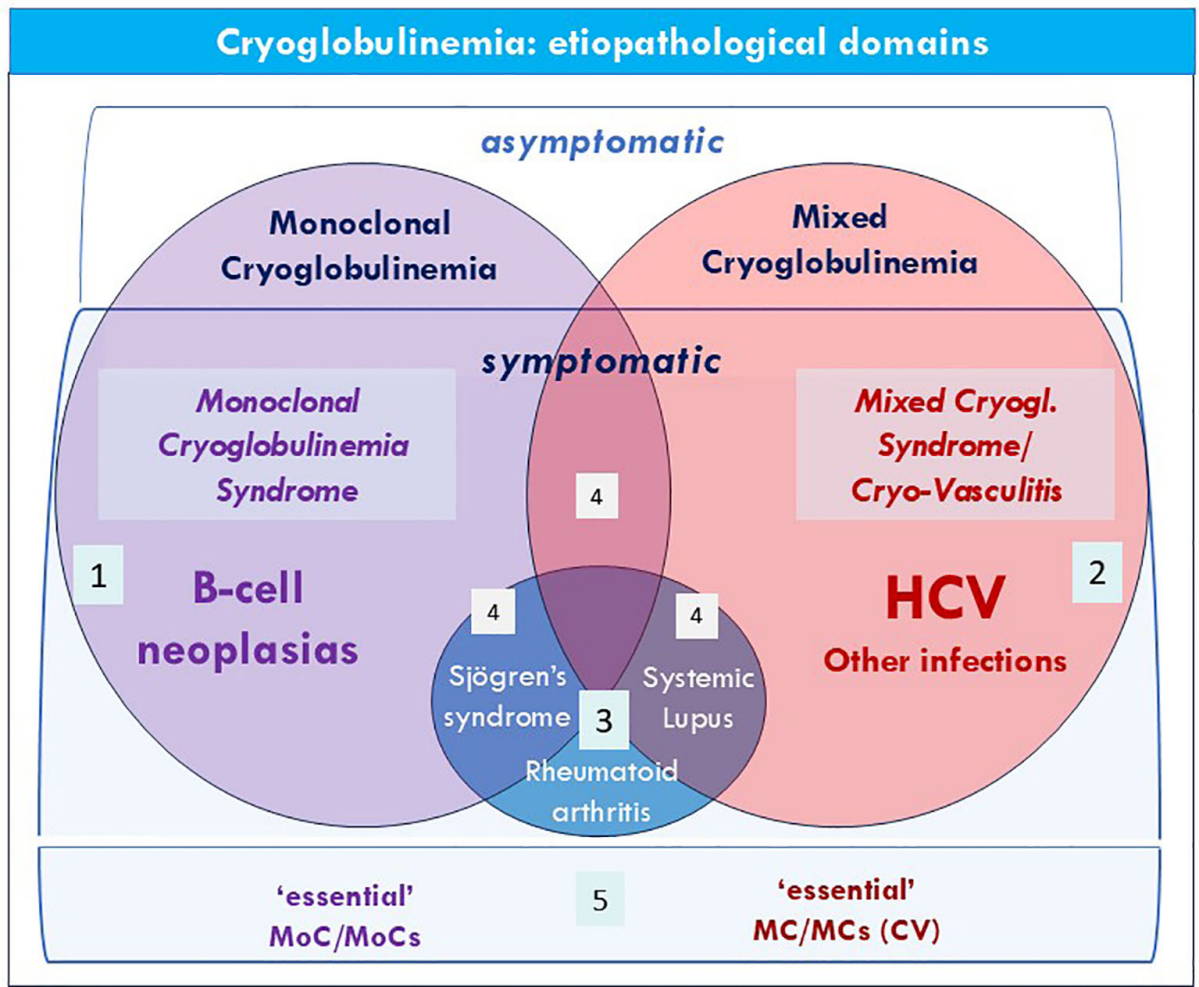
Cryoglobulinemia: etiopathological domains. The figure describes the main etiopathological domains that may be associated to monoclonal, type I, Cryoglobulinemia (MoC) or mixed, type II/III, Cryoglobulinemia (MC), with/without clinical manifestations, i.e. MoCs or MCs; 1. Hematological disorders, i.e. B-cell neoplasias (monoclonal gammopathy of indetermined significance; Waldenström’s macroglobulinemia, multiple myeloma, B-cell non-Hodgkin’s lymphoma, chronic lymphocytic leukemia); 2. Infectious diseases, especially HCV infection; 3. Autoimmune systemic diseases, such as Sjögren’s disease. systemic lupus erythematosus, rheumatoid arthritis. 4. There are overlapping areas among the three domains that may include individual patients with a variable combination of autoimmune, lymphoproliferative, and/or infectious manifestations. An example of this peculiar condition, not rare in clinical practice, is the coexistence in some patients of serum mixed cryoglobulinemia, ongoing HCV infection, and clinical symptoms, and laboratory alterations typical of both MCs and Sjögren’s disease, making their nosographic classification quite controversial (see text: 2 for differential diagnosis). A variable proportion of patients (top of the figure) with isolated serum cryoglobulins should be classified as ‘asymptomatic’ MoC or MC, needing careful clinical monitoring. 5. either MoC/MoCs or MC/MCs without any evidence of underlying hematological, autoimmune, and/or infectious conditions should be classified as ‘essential’ (see also **Table 1**).

According to Brouet and colleagues (4), cryoglobulinemia is traditionally classified into three immunological subtypes: type I, composed of a single monoclonal cryo-Ig (MoC), usually a paraprotein, types II and III mixed cryoglobulinemia (MC), characterised by polyclonal IgG and mono-oligo (type II) or polyclonal (type III) IgM RF, respectively. Type I, MoC is found mainly in the course of B-cell neoplasias (8, 9); while types II/III MC are frequently associated with different infectious, neoplastic, or immunological diseases (1–6, 10–13) (**Figure 2**).

With regards the types II/III MC that can be found in a variety of infectious diseases, we can hypothesize that they are the result of a chronic stimulation of the immune system by altered autologous IgG following the interaction with exogeneous factors, such as antigens of involved infectious agent(s). They may work as autoantigen able to elicit different cryo- and non-cryoprecipitable immune-complexes (IC), which in turn may be responsible for diffuse vasculitic lesions of mixed cryoglobulinemia syndrome (MCs) (1–6, 10–13).

## 2 Call for comprehensive nomenclature and definition of Cryoglobulinemias

The availability of shared nomenclature and definition systems is necessary to ensure unambiguous communication worldwide (nomenclature) and to establish specific features of a given disease and its boundaries (definition) towards other neighboring conditions, allowing uniform data collection and thus effective analysis. These latter are crucial either in the practical management of the disease and its clinical variants and for future research. Clinicians, researchers, and regulators can share information consistently within their own expertise as well across different medical disciplines. This is particularly relevant in the field of rare diseases, often characterized by high clinical-pathogenetic complexity and unpredictable outcomes, as for cryoglobulinemic syndromes.

Despite significant progress in recent decades regarding both disease etiopathogenesis and management (1–6, 10–13), the lack of a shared nomenclature and definition of cryoglobulinemia variants often leads to either patient selection and data collection quite inconsistent between different research centers, which may ultimately lead to poorly reliable results. Furthermore, a comprehensive nomenclature and definition of cryoglobulinemias may allow the definitive development of classification/diagnostic criteria for the main variants of cryoglobulinemia, i.e. MoC and MC. The vast literature available on the topic of ‘cryoglobulinemia’ (key word ‘cryoglobulinemia’ on PubMed: 5,261 results) offers a wide array of terms and definitions, most of which are correct *per se*, but potentially confusing, as noted above. These range from the generic term ‘cryoglobulinemia’, referring both to the simple *in vitro* phenomenon of cryoprecipitation and to the full-blown clinical syndrome, to ‘cryoglobulinemic disease’ instead of ‘cryoglobulinemia syndrome’, from ‘type I cryoglobulinemia’ as ‘monoclonal cryoglobulinemia’, from ‘essential’ or ‘idiopathic’ to ‘non-essential’ or ‘secondary’ cryoglobulinemia, to ‘cryoglobulinemic vasculitis’ synonymous with ‘mixed cryoglobulinemia syndrome’, and so on. Suitable and shared terminology and definitions are essential for developing classification/diagnostic criteria encompassing the numerous etiopathogenetic and clinical variants of cryoglobulinemia. Currently, classification criteria are available only for MCs; relating to this disorder, preliminary classification criteria were proposed by the Italian Group for the Study of Cryoglobulinaemias (GISC) back in 1989 (C Ferri et al. Proceedings of 10th Congress of the Italian Society of Immunology and Immunopathology, S. Margherita di Pula, Cagliari, 12 May, 1989), and subsequently revised and validated in multicenter GISC studies (14–16).

The present study, building on the currently used nomenclature, aims to propose a more orderly and comprehensive terminology and definition of the different cryoglobulinemia subtypes, with the primary goal of improving its applicability in routine clinical practice and facilitating the recruitment of homogeneous patient cohorts with distinct disease subtypes for clinical and etiopathogenetic research purposes.

## 3 Proposal of nomenclature and definition of cryoglobulinemias

As with any complex disorder, the development of nomenclature and definition of cyoglobulinemias is an ongoing process; it is necessarily based on the evolving knowledge of etiopathogenetic mechanisms with the indirect contribution of more recent clinical and therapeutic experiences. An updated nomenclature and definition of cryoglobulinemia subsettings is summarized in **Table 1**, which tray to rationalize all available terms, synonyms, and definitions often used but poorly suitable.

Their proper use may ensure the homogeneity of the cohorts studied and therefore the value of observed results; moreover, their refinement may contribute to the updating/development of classification/diagnostic criteria.

The first column reports the terminology of cryoglobulinemia subsettings as well their synonyms alongside (**Table 1**). In particular, the term ‘cryoglobulinemia’ should be reserved for the simple laboratory finding, i.e. the presence in the serum of one or more cryoprecipitable Igs (1–7). Similarly, either MoC or MC terms are specifically referred to only the presence of one monoclonal cryoprecipitable Ig or mixed Igs, more often IgG-IgM, respectively; in all cases regardless of the presence/absence of known causes and symptoms (**Table 1**, on the top). While the combination of serological (mono-/mixed cryoglobulins) and clinical features represents two distinct conditions, namely the MoC syndrome (MoCs) and the mixed cryoglobulinemia syndrome (MCs), which in turn may be termed ‘essential’ or ‘secondary’ according to the absence/presence of well-known causative factors or underlying disorders, respectively. The synonyms, type I and type II/III are referred to specific Ig composition of cryoprecipitates (1–7). Moreover, the term ‘cryoglobulinemic vasculitis’ is alternatively used to MCs; it focuses on the specific disease histopathological hallmark, i.e., leukocytoclastic vasculitis, for which the disease is traditionally classified among systemic vasculitis, in particular within the small vessel vasculitis subgroup (1–6, 17–19).

**Table 1:** Nomenclature and definition features of cryoglobulinemia subsetting.

Nomenclature & definition of cryoglobulinemia Sub-settings *clinical, serological, and pathological features, and disease domains*			
Nomenclature	Synonyms	Definition based on serogical, clinical, and pathological features	Disease domains*
*Cryoglobulinemia as laboratory finding*			
*Cryoglobulinemia*	_	This term should be referred to only the presence in the serum of one or more cryoprecipitable immunoglobulins, regardless of their composition and clinical features	Either with (*secondary) or* wihout (‘*essential)* evidence of hematological, autoimmune systemic diseases, and/or infectious disorders
*Monoclonal cryoglobulinemia (MoC)* *Mixed cryoglobulinemia (MC)*	Type I cryogl. Type II/III cryogl	These terms should be referred to only the presence in the serum of one cryoprecipitable monoclonal or mixed (IgG-IgM) immunoglobulins	
*Monoclonal Cryoglobulinemia syndrome*			
*Monoclonal Cryogl. Syndrome* Secondary to *’essential’ monoclonal* *cryoglobulinemia syndrome*	Type I cryogobulinemia s. Related/associated to ‘‘idiopathic” Type I cryogobulinemia s.	Serum monoclonal cryoglobulins (mostly IgG, less IgM, IgA) plus symptoms: fatigue, periph. neuropathy, skin lesions with non-inflammatory thrombotic lesions, hyperviscosity syndrome, symptoms of underlying disorders Idem, in the absence of other well-known hematological, autoimmune, and/or infectious disorders	B-cell lymphoproliferative disorders (MGUS, Waldenström’s m., MM, B-NHL, CLL, autoimmune systemic dis: Sjögren’s disease, SLE, RA, others and/or infectious disorders None
Mixed Cryoglobulinemia syndrome/Cryoglobulinemic Vasculitis			
*Mixed cryoglobulinemia* *syndrome* Secondary to *’essential’ mixed Cryogl. syndrome*	Cryogl. Vasculitis, Type II/III MCs Related/associated to *”idiopathic”* Cryogl. Vasculitis or Type II/III MCs	Serum mixed cryoglobulins, often low C4 and RF+, plus symptoms: weakness, arthralgias, purpura, peripheral neuropathy, multiple visceral organ inv. (deposits of IgG-M + complement at direct IF), skin leukocytoclastic vasculitis Idem, in the absence of other well-known hematological, autoimmune, and/or infectious disorders	HCV infection, other infections: HBV, HIV, EBV, PV-B19, herpes, varicella–zoster, human T-cell leukemia type 1, influenza, and rubella virus, etc.; toxic agents Rare hematological/rheumatic-autoimmune disorders None

*disease domains: hematological, rheumatic-autoimmune, and/or infectious disorders.

Hematological dis.: monoclonal gammopathy of clinical significance (MGUS), Waldenström’s macroglobulinemia, multiple myeloma (MM), non-Hodgkin’s lymphoma (NHL), chronic lymphocytic leukemia (CLL).

Autoimmune systemic dis.: SLE, systemic lupus erithematosus; RA, rheumatoid arthritis.

HCV, hepatitis C virus; HBV, hepatitis B virus; HIV, human immunodeficiency virus; EBV, Epstein-Barr virus; PV-B19, parvovirus B19.

The definition of both MoCs and MCs is mainly based on different serological, clinical, and pathological features; they are commonly associated with various pre-malignant, malignant, infectious, or autoimmune conditions, which in turn represent the underlying cause for the cryoglobulin production (1–9). With regards to the disease domains involved in cryoglobulinemia variants (**Figure 2**, **Table 1**, column on the right), both MoCs and MCs may share the same hematological, rheumatic-autoimmune, and/or infectious disorders, with a marked prevalence of lymphoid neoplasms for MoC and infectious diseases, mainly HCV infection, for MC (1–6, 17, 18).

## 4 Monoclonal cryoglobulinemia syndrome

The serological hallmark of MoC is the presence of a single monoclonal cryoprecipitable Ig (mainly IgG or IgM; **Table 2**) produced by monoclonal expansion of B-cell clone as expression of different lymphoid neoplasms, namely indolent monoclonal gammopathy of unknown significance (MGUS), smoldering Waldenström’s macroglobulinemia, or overt malignant disorders such as multiple myeloma (MM), B-cell non-Hodgkin’s lymphoma (B-NHL), or chronic lymphocytic leukemia (CLL) (20). The two distinctive features best defining the MoC are the very frequent association with hematologic neoplasias (9) and the peculiar pathological alterations responsible for the clinical syndrome (1, 5, 17, 18) (**Table 3**). In a large series of 102 MoC patients (8), the presence of symptoms (MoCs) was observed in 87% of cases; the most common were cutaneous (total 63%, with purpura 42% and ulcers/gangrene 34%) and neurological manifestations (32% of cases), most frequently sensory neuropathy (24%), Raynaud’s phenomenon (25%), arthralgias (24%), and renal manifestations, mainly glomerulonephritis (14%). Of note, the large majority of MoC patients had an underlying lymphoproliferative disorder (92%), while essential MoC was present in only a small percentage of patients (8). The symptoms of MoCs are the consequence of intravascular cryoprecipitation and deposition of monoclonal cryo-immunoglobulin responsible for non-inflammatory vessel occlusion and ischemic tissue damage. In some individuals the large amount of cryoglobulins, not rarely presenting as cryogel in isolated serum samples, are responsible for *in vivo* marked increase of blood viscosity with overt hyperviscosity syndrome (8, 9, 18, 21). This harmful complication is characterized by neurological symptoms (headache, confusion, tinnitus, vertigo, confusion, ataxia, and coma), blurry vision or vision loss, retinal hemorrhages and venous “sausaging”, sudden deafness, epistaxis, necrotic lesions of acral districts (21–25). In anecdotal observations monoclonal cryoglobulins associated to MM or other lymphoid neoplasms, even at quite low concentrations may form various morphologies of cryoglobulin condensates from different patients, including crystals, amorphous aggregates, and gels (26, 27). The intravascular precipitation of MoC appears *in vivo* as hialin thrombi occluding small blood vessels, more often in endoneural microvessels (20) responsible for severe peripheral neuropathy as well in the renal glomeruli as massive intraluminal cryoprecipitation with rapidly progressive renal failure; the latter finding characterizes patients with so-called monoclonal gammopathy of renal significance (28).

**Table 2:** Cryoglobulinemia & autoimmune systemic diseases: differential diagnosis.

Cryoglobulinemia & autoimmune systemic diseases: differential diagnosis					
Clinical, laboratory & pathological features	Mono-cryogl. syndrome	Mixed cryogl. syndrome	Sjögren’s disease	Systemic lupus erythematosus	Rheumatoid arthritis
Clinical					
Purpura-weakness-arthralgias	+	+++	++	+	+
Sicca syndrome	+*	+*	+++	+*	+*-
Skin Ulcers	++	+++	+	+	+
Arthritis	-/+	-/+	+	+	+++
Liver inv.	-	++	+	-	-
Renal inv**	+	++	+/-	++	-
Lung inv	-	+	++	+	+
Peripheral neuropathy	++	+++	++	+/-	+/-
Hyperviscosity syndrome	++	-/+	+	-	-
Laboratory					
Cryo. Composition	Type I	Type II-III	Type I-II	Type I/II-III (rare)	Type I/II-III (rare)
Low C3	-	-/+ rare	-	++	-
Low C4	-	+++	-	++	-
RF/ACPA	±/-	+++/-	++/-	+/-	++/++
ANA+	-/+	-/+	+++	+++	+
Anti-ENA+	-	-/+	+++ (SSA-Ro/SSB-La)	+++ (anti-dsDNA, -Sm)	+
Anti-dsDNA	-	-	-/+	+++	-
Pathological					
Vascular occlusion	++	-	-/+	-	-
Immune-complex vasulitis	-	+++ (MC + C)	-/+	+++ (IC + C)	-/+
Leukocytoclastic vasculitis	-	+++	+/-	-	-
IC+Complement deposits	-	+++	-/+	+++	-/+
Underlying B-cell neoplasia	+++	+	+	rare	rare

*generally mild symptom with mild/absent typical histopathological findings, and specific autoantibodies.

**membranoproliferative glomerulonephritis; RF, rheumatoid factor; ACPA, anti-citrullinated protein antibodies; ANA, antinuclear antibodies; anti-ENA, anti-extractable nuclear antigen antibodies; IC, immunocomplex; C, complement.

**Table 3:** Summarizes the main clinical and serological features of monoclonal and mixed cryoglobulinemia syndromes, stratified in essential and secondary forms and the main underlying disorders.

Summary of the main clinical-serological features of different types of cryoglobulinemia				
	*Monoclonal cryoglobulinemia syndrome*		*Mixed cryoglobulinemia syndrome/cryoglobulinemic vasculitis*	
Feature	essential	secondary	essential	secondary
*Clinical*	fatigue, peripheral neuropathy, non-inflammatory thrombotic skin lesions, hyperviscosity syndrome, symptoms of underlying disorders	idem	purpura, weakness, arthralgias, peripheral neuropathy, multiple visceral organ inv. (deposits of IgG-M + complement at direct IF), skin leukocytoclastic vasculitis	idem
*Serological*	monoclonal cryoglobulins (mostly IgG, less IgM, IgA)	idem	mixed IgG-IgM cryoglobulins, low C4, rheumatoid factor	Idem
*Underlying disorders*	none	B-cell lymphoproliferative, autoimmune, and/or infectious diseases*	none	HCV infection, others; toxic agents, B-cell lymphoproliferative, autoimmune, and/or infectious diseases*

## 5 Mixed cryoglobulinemia syndrome, cryoglobulinemic vasculitis

The disease, first described by Meltzer et al. in 1966 (2), as ‘essential’ Mixed cryoglobulinemia syndrome (MCs), was classified as autonomous disorder when other well-known immunological, infectious, or neoplastic conditions had been ruled out. Besides the laboratory hallmark, i.e. serum mixed cryoglobulins, very often associated with low complement C4, other distinctive pathological features of MCs are the skin leukocytoclastic vasculitis with frequent multiple organ involvement (1–6, 10–12, 14–17, 29–31). Therefore, the disease is also termed ‘cryoglobulinemic vasculitis’, and classified among systemic small vessel vasculitides, which also includes cutaneous leucocytoclastic vasculitis and Schonlein-Henoch purpura (19).

MCs is clinically characterized be the triad—purpura, weakness, arthralgias— (**Table 2**) and by numerous organ involvement; namely, membranoproliferative glomerulonephritis, peripheral neuropathy, skin ulcers, diffuse vasculitis, and less frequently neoplastic complications, mainly lymphatic malignancies (2–6, 10–17, 29–31). The prevalence of MCs shows a great geographical heterogeneity; it is more common in Southern than in Northern Europe or North America. This is particularly true for MCs associated with hepatitis C virus (HCV) infection which is also much more diffuse in the same some geographical areas such as the Mediterranean basin countries (5, 6, 10, 32).

### 5.1 MCs and HCV infection

With regards the etiopathogenesis of MCs, the presence of chronic hepatitis among the most frequent manifestations suggested a possible role for hepatotropic viruses in the pathogenesis of the disease (1–4). Since the 1970s a causative role of hepatitis B virus (HBV) has been investigated; however, this virus may be involved in only a minority of MCs patients (1, 5, 6, 33, 34). On the contrary, ongoing HCV infection has been firstly demonstrated by polymerase chain reaction technique in the majority of patients since 1991 (35), and successively confirmed in various MCs patients’ series worldwide (1, 6, 10, 30, 31, 33, 34, 36–39). Therefore, over three-quarters of MCs was classified as ‘secondary’, i.e. HCV-related MCs (30, 31, 34, 35, 37). With the introduction of direct-acting antivirals (DAAs), able to eradicate HCV in a very high percentage of individuals, a progressive, marked reduction of HCV-related cases and consequently of the whole MCs patients population was observed, especially in the most developed countries thanks to the greater diffusion of these new antivirals (18, 36, 40).

### 5.2 ‘HCV syndrome’

The study of the association between HCV and MCs in a high percentage of patients prompted a series of investigations, leading to important results such as the identification of the HCV lymphotropism responsible for B-cell lymphoproliferation (41, 42) together with its oncogenetic role in lymphoid malignancies (1, 10, 43–45). In patients with HCV infection, the interaction between this virus and the host immune system, as well as its direct oncogenetic role, can lead to the development of different clinical phenotypes in a significant number of subjects (1, 10). In addition to HCV-related MCs, a broad spectrum of autoimmune disorders and malignancies can be observed in HCV-infected individuals with highly variable frequencies in various populations worldwide based on different genetic and/or environmental predisposing factors (1, 5, 10, 43, 46).

In this scenario, HCV-related MCs represents a crossroads between possible organ- and non-organ-specific autoimmune disorders and neoplasias, especially B-cell non-Hodgkin’s lymphoma (1, 5, 10, 43, 44, 46, 47). The complex of these disorders observed in the course of HCV infection has suggested the term ‘HCV syndrome’ (10).

Apart from the marked decrease in HCV infection already observed in developed countries—and desirable worldwide—the HCV-related syndrome represents a useful model for investigating the etiopathogenesis of other virus-driven autoimmune and lymphoproliferative/neoplastic disorders (10).

## 6 Cryoglobulinemia and autoimmune systemic disorders

The term ‘‘essential’’ is referred to both MoCs and MCs as autonomous conditions when a number of well-known systemic autoimmune, infectious, or neoplastic disorders had been ruled out by careful patient evaluation (1–4, 6, 8, 10) (**Table 2**). However, in some individuals a definite diagnosis remains very difficult because of their clinical polymorphism. Clinically, both MoCs and MCs may share a number of symptoms with other autoimmune systemic diseases, particularly with primary Sjögren’s disease, rheumatoid arthritis (RA), and Systemic Lupus erythematosus (SLE) (1, 5, 10)(**Table 2**). Sicca syndrome may be recorded in about half of MCs patients, even if the current classification criteria for primary Sjögren’s disease are satisfied in very few cases (1, 5, 10, 47). Moreover, both primary Sjögren’s disease and MCs may share many symptoms, such as xerostomia and/or xerophthalmia, purpura, arthralgias, RF seropositivity, serum cryoglobulins, and less frequently B-cell lymphoma (1, 5, 10, 47). In these instances, a careful patient clinical assessment may be sufficient for a correct diagnosis on the basis of the following distinctive findings: primary Sjögren’s disease shows specific autoantibody patterns (anti-RoSSA/LaSSB) and histopathological alterations of salivary glands, which are rarely found in MCs; on the contrary, cutaneous leukocytoclastic vasculitis and visceral organ involvement, such as immune-complex-mediated membranoproloferative glomerulonephritis type I (MPGN type I) are typically found in MCs ( 2). In the few cases in which the differential diagnosis may result very difficult, it might be appropriate to classify this condition as overlapping MCs- primary Sjögren’s disease. This condition is often characterized by low rate of serum anti-RoSSA/LaSSB along with increased prevalence of hypocomplementemia, monoclonal/mixed cryoglobulinemia, other autoimmune-lymphoproliferative manifestations, mainly B-cell lymphomas, and overall more severe outcome (48), Hovewer, the risk of malignant lymphoma is significantly increased in cryoglobulinemic vasculitis patients compared to those with Sjögren’s disease, benign B-cell proliferation, and cutaneous hypergammaglobulinaemic vasculitis as reported by Quartuccio et al. (49) and more recently by Breillat et al. (50).

From a practical point of view, the overlapping MCs-primary Sjögren’s disease might be better managed as vasculitic disorder considering its therapeutic and prognostic implications (10).

In MCs patients we can observe the presence of symmetrical, erosive, rheumatoid-like polyarthritis resulting in an overlapping MCs/RA syndrome. In these cases, the detection of serum anti-cyclic citrullinated peptide antibodies, markers of classical seropositive RA, may be a useful tool for differential diagnosis (5, 10). A similar condition can be observed in patients with concomitance of MoCs or MCs and SLE; some MCs characteristics, such as purpuric lesions of the lower limbs with classical leukocytoclastic vasculitis, MPGN type I, and isolated low C4, from one side, and typical histologic pattern of SLE glomerulonephritis, with low levels of both C3 and C4, anti-dsDNA, ANA and anti-ENA seropositivity from the other side, are generally sufficient to differentiate these conditions (5, 10).

Finally, very high levels of cryocrit responsible for clinically overt hyperviscosity syndrome may complicate MoCs; this severe complication is very rare in the course of MCs as well other autoimmune systemic disorders (1, 10, 20, 21, 23–28, 51).

## 7 Assessment of patient with cryoglobulinemia

Cryoglobulinemia is a frequent laboratory finding, very often without any clinical relevance. Low serum levels of MoC or MC are detectable in a wide number of acute or chronic infections, as well in autoimmune or lymphoproliferative/neoplastic diseases (1–6).

**Figure 3:**
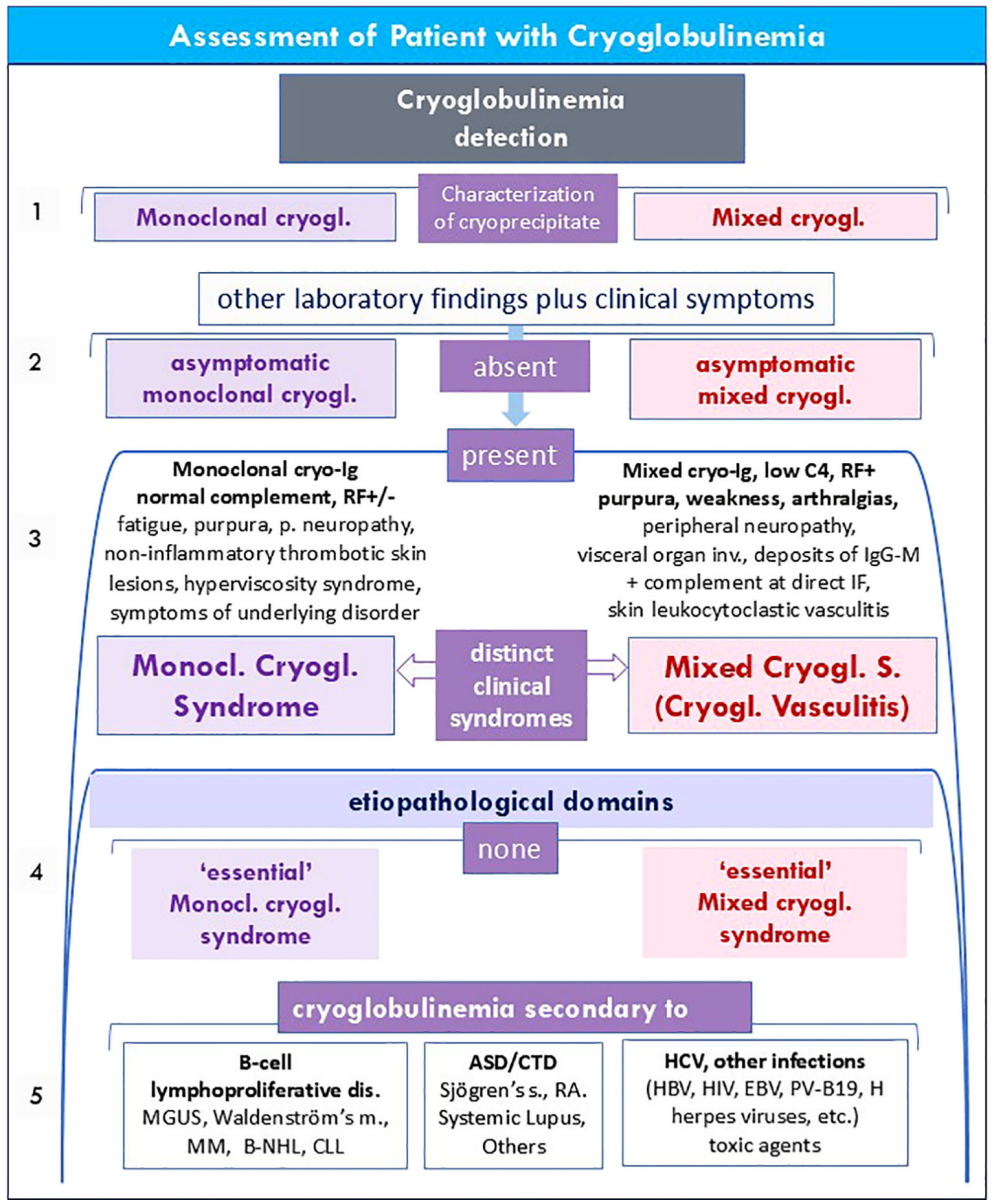
Assessment of patients with cryoglobulinemia. Serum cryoglobulins may be detected as incidental laboratory finding or because suspected in patients with typical cryoglobulinemic manifestations, namely clinical triad -purpura, weakness, arthralgia-, dyschromic orthostatic lesions, sock-like paresthesias in the lower limbs, and/or skin ulcers with/without gangrene, and/or immunolopgical alterations (rheumatoid factor seropositivity and/or low C4), clinical manifestations of lymphoid neoplasms, autoimmune systemic disorders (ASD) and/or infections (HCV, HBV, etc). 1. Following the immunological characterization of cryoprecipitate (see also **Figure 1**), the cryoglobulinemia is classified as type I MoC or type II/III MC. 2. Both disorders can be classified as ‘asymptomatic’ or 3. ‘symptomatic’ (MoCs or MCs/cyoglobulinemic vasculitis) according to the absence/presence of typical cryoglobuinemic manifestations. 4. either mono- and mixed cryogl. syndromes should be classified as ‘essential’ in the absence of underlying etiopathological disorders or 5. ‘secondary’ to other well-known conditions within three main etiopathological domains; namely, B-cell lymphoproliferative disorders (more often for the monoclonal cryoglobulinemic syndrome), infectious diseases (especially for HCV-related MCs), and ASD (both MoC and MC) when in overlap with Sjögren’s disease, systemic lupus erithematosus, rheumatoid arthritis (RA), etc.

For a correct evaluation of a patient with cryoglobulinemia the first steps can be crucial (**Figure 1**, **3**). In a subject with either occasional cryoglobulin detection or clinical manifestations suggesting a cryoglobulinemic syndrome, it is necessary to firstly evaluate the immunological composition of the cryoprecipitate, i.e., mono-/mixed (type II/III) cryoglobulinemia (1, 5, 37). At the same time, a careful recording of typical cryoglobulinemia signs and symptoms should be performed in order to classify the serum MoC or MC as asymptomatic or symptomatic (**Figure 3**, points 1 & 2; **Figure 1**, **Figure 3**). As regards cryoglobulin detection, some simple precautions are necessary to avoid false negative/positive results. In particular, the first procedures, i.e. blood sampling, clotting, and serum separation by centrifugation, should invariably be performed at 37 °C, while the cryocrit determination and cryoglobulin characterization should always done at 4 °C, after seven days from the serum isolation (1, 5, 37) (**Figure 1**). Finally, the cryocrit determination should be done on blood samples without anticoagulation in order to exclude false positive results caused by cryofibrinogenemia (52). Since Ig cold precipitation may be an intermittent phenomenon, it is not rare to observe individuals with typical manifestations of cryoglobulinemia syndrome but without serum cryoglobulins at the first determination; therefore, in these cases periodic re-evaluations are appropriate (1, 5, 37). This particular condition is possibly due to an early Ig precipitation immediately after blood sampling at room temperature, considering that the temperature range (between 4° and 37 °C) and the rapidity of cryoprecipitation are very wide. Alternatively, it may be the expression of the wide variability in the percentage of cryoprecipitable immune-complexes observable either among patients and in single cases during the course of the disease (1, 5, 33, 37, 53).

After isolating and washing the cryoprecipitate, the identification of cryoglobulin components can be carried out by immuno-electrophoresis or immunofixation; these analyses must be performed always at 37 °C to avoid cryoglobulin precipitation/loss during the procedures (1, 5, 37).

Serum cryoglobulin levels, namely cryocrit percent, usually do not correlate with the severity and prognosis of the disease (1, 5). Particularly low cryocrit values may be observed also in severe MCs; while, high serum cryoglobulin concentrations may characterise a paucisymptomatic disease course, with the exception of individuals developing a classical hyperviscosity syndrome, in some cases with a cryogel phenomenon, responsible for severe rheological manifestations This harmful condition may be found in patients with MoCs but very rarely in MCs (1, 5, 37).

In patients with symptomatic cryoglobulinemia (**Figure 3**, point 3), the definition of MoCs or MCs should be based on some peculiar findings that characterize these distinct disorders. In particular, MoCs may show non-inflammatory thrombotic skin lesions and/or overt hyperviscosity syndrome, while other symptoms such as weakness, purpura, and especially peripheral neuropathy are frequently recorded in both disorders (1, 5, 18, 37). On the other hand, MCs is characterized by purpuric skin lesions secondary to typical leukocytoclastic vasculitis and frequent visceral organ involvement, with tissue deposits of IgG-M immune-complexes and complement at direct IF, as typically found in the cryoglobulienemic membrano-prolipherative glomerulonephritis type I (1, 5, 18, 33, 37).

Finally, for a comprehensive assessment of patients with MoCs or MCs it is important to define the etiopathological domains in which to frame these two disorders. The major disease domains underlying MoCs or MCs are: i. the B-cell lymphoproliferative diseases (Waldenström’s macroglobulinemia, multiple myeloma, B-cell non-Hodgkin lymphoma, chronic lymphocytic leukemia, and monoclonal gammopathy), ii. some autoimmune systemic disorders, among which the primary Sjögren’s disease, rheumatoid arthritis, and systemic lupus erythematosus, and iii. infectious and toxic agents, more frequently the hepatitis C virus infection (1, 5, 18, 33, 37) (**Figure 2**). In the absence of underlying well-defined disorder, both MoCs or MCs should be defined as ‘essential’. A careful clinical and serological screening of cryoglobulienemic patients is necessary in all patients, at the referral and at periodic time intervals, especially in the case of rapid onset atypical clinical manifestations, especially given the important therapeutic implications (1, 5, 18, 33, 37).

## 8 Monoclonal and mixed cryoglobulinemia syndromes: two distinct disorders

The clinical syndromes associated to the presence of serum monoclonal or mixed cryoglobulinemia, i.e. MoCs and MCs, must be regarded as two distinct disorders, even if they may share a number of immune-lymphoproliferative and clinical similarities (1, 4, 5, 18) (**Tables 2**, **3**). A part from the rare cases of ‘essential’ MoCs, the presence of underlying lymphoid malignancy is particularly common in MoCs and is instead a late event, much less frequent in MCs (1, 8, 9). The latter is often supported by a low-grade, indolent B-cell lymphoma (8, 9). Moreover, MoCs is less frequently associated with chronic infections or ASD (8, 9), while MCs may be triggered by numerous infectious agents, mainly HCV, in a significant proportion of individuals, and sometimes present in ASD, particularly the Sjögren’s disease (1, 5, 10) (**Table 2**).

### 8.1 Differential diagnosis

Considering that MoCs and MCs share numerous clinical features, in some patients the differential diagnosis my result quite difficult. In particular, constitutional symptoms (weakness, fatigue, weight loss), peripheral neuropathy, skin lesions, and sicca syndrome are commonly recorded in both disorders. Contrarily, the hyperviscosity syndrome is one of the most frightening complications of MoCs but very rare in MCs (**Tables 3**, **4**, **Figure 3**).

**Table 4:** Etiopathogenesis, clinical features & treatment of cryoglobulinemias.

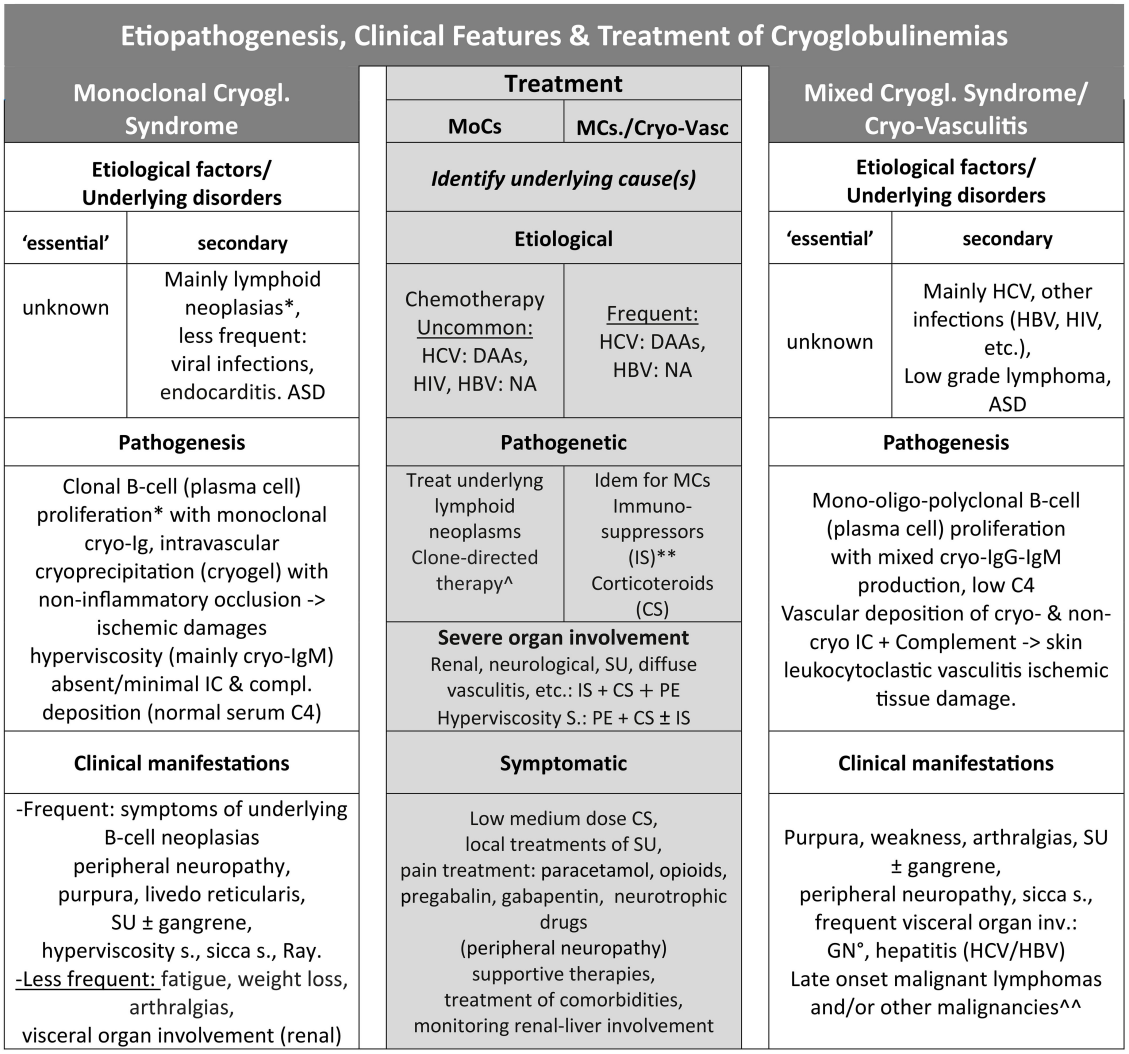

MoCs, monoclonal cryoglobulinemia syndrome; MCs, mixed cryoglobulinemia syndrome; *Waldenström’s macroglobulinemia, multiple myeloma, B-cell non-Hodgkin’s lymphoma (B-NHL), chronic lymphocytic leukemia, monoclonal gammopathy of indetermined significance.

**Immunosuppressors (IS), rituximab, cyclophosphamide, azathioprine, methotrexate, bortezomib (in overlap with ASD), chemotherapy for lymphoid malignancies (DAAs, direct acting antivirals; NA, nucleos(t)ide analogues: entecavir, tenofovir, etc. Therapies for underlying lymphoproliferative disorders or ASD Immunomodulator/suppressors; Corticosteroids (CS); PE, Plasma exchange; IC, immunocomplexes; SD, autoimmune systemic diseases; °GN, membranoproliferative glomerulonephritis type I; Ray, Raynaud’s phenomenon; ^: papillary thyroid cancer, hepatocellular carcinoma (HCV-associated).

The immunological hallmarks, i.e. serum monoclonal and mixed cryoglobulins, respectively, are sufficient in the majority of cases to correctly classify these disorders (1, 5, 10). Hovewer, in the clinical practice there is a grey zone in rare cases it is possible to observe the concomitancy of both serotypes (1, 5, 8–10). Other distinctive features are the specific microvascular alterations detectable by histopathological examination of skin lesions, namely the non-inflammatory thrombotic manifestations that characterize the MoCs, on one side, and the cryo-/non-cryoprecipitable immune-complex-mediated vasculitis on the other side (1–6, 8, 9, 18). Therefore, a correct immune-pathological and clinical assessment of cryoglobulinemic patients is mandatory (**Figure 3**). Hovewer, in clinical practice there is a gray area (**Figure 2**) encompassing some individuals that can be classified within all etiopathogenetic domains due to the concomitance of multiple alterations that ultimately are very difficult for an overall definition. An example is the possible observation of a patient with underlying B-cell lymphoma and pathological, immunological, and clinical manifestations fulfilling the diagnosis of both Sjögren’s disease and MCs. This peculiar symptom complex can become even more intriguing due to the additional presence of HCV infection and in rare cases of both mono-/mixed cryoglobulin serotypes (1, 5, 6, 10).

A rational therapeutic approach to such cases is to carefully evaluate the clinical manifestations by prioritizing them according to their severity and prognostic value.

Overall, the above etiopathogenetic and clinical differences among MoCs and MCs should drive either the treatment strategies, tailored on individual cases, and the long-term patient monitoring. In addition, the treatment of cryoglobulinemia variants should be based on three different levels (**Table 4**): i. etiological therapy should always be considered in the presence of a clear causative agent; ii. more often, pathogenetic therapy is able to counteract the frequent underlying disorders or pathogenetic mechanisms responsible for organ damage in both MoCs and MCs; and iii. symptomatic and supportive therapies are invariably necessary for the numerous clinical manifestations. Finally, the combination of different therapeutic typologies is essential in clinical practice.

### 8.2 Treatment of monoclonal cryoglobulinemia syndrome

The first step of therapeutic approach to these patients is to identify the possible underlying cause(s) of the disease (**Tables 3**, **4**, **Figure 2**); in particular, the type and prognostic value of the lymphoid neoplasias given their stringent association with MoCs (8, 9). Therefore, the first choice ‘etiological’ treatment is mainly directed at the possible presence of some lymphoid malignancies such as MM, B-NHL, or CLL, as the association with viral infections is anecdotal (8, 54–56). Hyperviscosity syndrome is frequently observed in MoCs, especially when associated with Waldenström’s macroglobulinemia with markedly elevated IgM (1, 8–10, 20, 21, 23–28, 51). Timely introduction of pathogenetic therapy is necessary for this medical emergency, mainly based on plasmapheresis, possibly with corticosteroids, for the rapid reduction of serum viscosity (24–26, 51). In some cases, chemotherapy directed at underlying lymphoid neoplasia may be appropriately associated (57–59).

Pathogenetic/symptomatic treatments are frequently required for different MoCs clinical manifestations, with particular attention to neuropathic pain. Finally, supportive therapies and treatment of comorbidities are highly advisable (57–59) (**Table 4**).

### 8.3 Treatment of ‘essential’ MCs/cryoglobulinemic vasculitis

Since MCs and MoC share a number of etiopathogenetic and clinical manifestations, the therapeutic approach to ‘essential’ MCs includes many of the treatments mentioned above.

In very rare cases both cryoglobulinemia variants, i.e. MCs and MoC, may be detected in the same patient (60). Therapeutic strategies of ‘essential’ MCs are fundamentally directed at the underlying B-cell neoplasm especially in the few cases in which the lymphoproliferative disorder is clinically fearful, such as the case of overt B-cell non-Hodgkin’s lymphoma or other B-cell malignancies. In most patients with low-grade, indolent lymphoma, the treatment primarily aims to manage the pathogenic autoimmune/inflammatory component responsible for vasculitic manifestations, with the use of immunomodulatory/immunosuppressive drugs and corticosteroids, preferably tailored to the individual patient (**Table 4**).

In the case of particularly aggressive clinical manifestations, i.e. rapidly progressive glomerulonephritis, severe sensory/motor peripheral neuropathy, widespread vasculitis, and/or hyperviscosity syndrome (rare) the combination of plasmapheresis, high dose steroids, cyclophosphamide (or rituximab), and/or chemotherapy (60, 61) should be undertaken promptly. Many MCs patients are paucisymptomatic for long time periods; therefore low dose steroids (6-methylprednisolone 2–8 mg/day) and supportive therapies may be sufficient to control symptoms (purpura, arthralgias, fatigue, etc.); in these subjects a careful patient’s monitoring is mandatory in order to timely diagnose sudden onset of worse MC complications (**Table 4**).

### 8.4 Treatment of MCs/cryoglobulinemic vasculitis ‘secondary’ to HCV-/HBV infection

#### 8.4.1 HCV-related MCs

The therapeutic management of MCs associated with chronic hepatitis C virus (HCV) infection has undergone a radical transformation over the past decade. Historically, interferon (IFN)-based regimens were the only available antiviral approach, yet their utility was limited by poor tolerability, contraindications in patients with advanced systemic vasculitis, and inconsistent results in terms of clinical remission (18) (**Table 4**). The introduction of direct-acting antivirals (DAAs) has profoundly changed this scenario, offering both a safer and far more effective strategy for viral eradication. Numerous clinical studies have demonstrated that IFN-free DAA combinations achieve sustained virological response (SVR) rates exceeding 90% in patients with cryoglobulinemic vasculitis, comparable to outcomes in those without extrahepatic manifestations. Viral eradication is closely associated with the regression of vasculitic symptoms, including purpura, arthralgia, and weakness, which typically improve during or shortly after therapy. However, improvement in more severe organ involvement, particularly neuropathy and renal disease, may occur more slowly or remain incomplete (36, 62).

Despite the impressive efficacy of DAAs, 10–20% of patients experience persistent or recurrent cryoglobulinemic activity even after successful HCV clearance. These cases underscore the multifactorial nature of cryoglobulinemic vasculitis, in which immune dysregulation and clonal B-cell expansion may persist independently of the viral stimulus (40, 63, 64). In such situations, adjunctive immunotherapy becomes essential. The anti-CD20 monoclonal antibody rituximab (RTX) has emerged as the most effective option for controlling persistent vasculitis, either alone or combined with glucocorticoids or plasma exchange (65). Its use has consistently produced durable clinical and immunological responses with an acceptable safety profile, even in patients with advanced liver disease (65–67). RTX may also reverse hepatic inflammation and improve liver function, likely through indirect immunomodulatory effects (68). Corticosteroids and colchicine are used as supportive measures to control inflammatory activity, while plasma exchange provides rapid removal of circulating immune complexes in patients with severe vasculitis, renal failure, or extensive skin necrosis (69) (**Table 4**).

Long-term observational studies have highlighted several predictors of incomplete response or relapse after DAA therapy, including older age, baseline renal involvement, high rheumatoid factor titers, and evidence of residual B-cell clonality such as t (14, 18) translocation or Notch4 gene polymorphisms (40, 64, 70). Flares may also occur in association with conditions that trigger intense immune activation, including severe infections or vaccinations such as those against influenza or SARS-CoV-2, though vaccine-related episodes are typically mild and self-limiting (71). Therefore, the current therapeutic paradigm for HCV-MCs is a stepwise, multidisciplinary approach: first, prompt and complete viral eradication using DAAs; second, individualized immunologic modulation, most commonly through rituximab-based therapy, to control or prevent relapses and long-term organ damage.

#### 8.4.2 HBV-related MCs treatment

Cryoglobulinemic vasculitis secondary to chronic hepatitis B virus (HBV) infection represents a rarer but clinically relevant entity, requiring a distinct management strategy. In contrast to HCV-related disease, where antiviral therapy may be time-limited, treatment of HBV-associated vasculitis focuses on the long-term suppression of viral replication rather than true viral eradication. The backbone of therapy is nucleos(t)ide analogue (NA)–based antiviral treatment, which reliably suppresses HBV DNA replication, leading to a marked improvement in systemic manifestations such as purpura, fatigue, and arthralgia in most patients (36, 72, 73) (**Table 4**). Within 6–12 months of continuous therapy with agents such as entecavir or tenofovir, up to 80% of patients experience partial or complete remission of vasculitic symptoms (74). In contrast, interferon-based regimens are now largely abandoned due to their limited tolerability and risk of exacerbating immune-mediated vascular injury.

Patients presenting severe or refractory manifestations, such as skin ulcers, motor neuropathy, or glomerulonephritis, may benefit from adjunctive therapies including corticosteroids, plasma exchange, or rituximab. The latter has proven particularly effective in controlling systemic inflammation and B-cell hyperactivity; however, its use requires careful virological monitoring due to the potential for HBV reactivation. For this reason, co-administration of a potent antiviral agent is mandatory when RTX is prescribed (33). When combined appropriately, RTX and NAs can provide substantial clinical improvement even in advanced cases. Importantly, long-term continuation of antiviral therapy is essential, as withdrawal may result in HBV reactivation and recurrence of cryoglobulinemic vasculitis. Han et al. (75)described a relapse following the discontinuation of entecavir, which resolved upon re-initiation of treatment, illustrating the need for sustained viral suppression.

From an immunological perspective, antiviral therapy with NAs leads to a reduction in cryocrit levels and rheumatoid factor activity in most patients, while complement (C4) levels tend to rise more gradually and may remain below normal. Despite the frequent need for prolonged or even lifelong antiviral therapy, this strategy remains the most effective and safest means of controlling both hepatic disease and systemic vasculitis. In selected cases, combined antiviral and immunomodulatory therapy achieves not only clinical remission but also stabilization of renal function and prevention of further organ damage. The integration of virological control with tailored immunosuppression thus represents the cornerstone of contemporary management for HBV-related cryoglobulinemic vasculitis.

## 9 Conclusion

Cryoglobulinemia refers to the mere presence of serum cryo-Ig. This phenomenon is a relatively frequent laboratory finding observable even in apparently healthy individuals, often transient and devoid of clinical significance. In many cases, it may remain asymptomatic; however, its clinical significance is highly context-dependent and should not be underestimated. Only in a limited number of cases does this peculiar *in vitro* phenomenon represent a true pathological condition, which may in turn conceal an underlying lymphoproliferative/neoplastic, autoimmune, and/or infectious disorder. In current usage, the term cryoglobulinemia is often employed interchangeably to denote both the innocent presence of serum cryoglobulin precipitation *(*MoC or MC*)* and the associated clinical syndromes *(*MoCs or MCs*).* Therefore, the term “cryoglobulinemia” alone is inadequate for the proper definition of cryoglobulinemic patients. For nosographic, diagnostic, and especially clinical reasons, a universally accepted nomenclature and definition are required. To achieve this, a series of straightforward evaluative steps should be followed. First, the detection of serum cryoglobulins, whether incidental or prompted by suggestive clinical manifestations, must always be followed by isolation and immunologic characterization of the cryoprecipitate as either MoC or MC, along with a comprehensive clinical assessment of the patient and a search for potential underlying or associated disorders, even in apparently asymptomatic cases. In particular, immune-serological, microbiological, and pathological investigations are essential to fully classify both MoC and MC, distinguishing between asymptomatic and symptomatic forms, and between essential and secondary forms when associated with one or more of the aforementioned disease domains.

Patients with asymptomatic serum MoC or MC require careful longitudinal monitoring, both for the potential progression to overt clinical syndromes *(*MoCs or MCs), as well as those with essential variants for the development of underlying disorder(s). The latter, when present, represent the primary therapeutic challenge, as in the case of B-cell malignancies, HCV or HBV infection, and/or systemic autoimmune diseases such as Sjögren’s disease.

The two main clinical entities, MoCs and MCs (also termed cryoglobulinemic vasculitis), share a wide spectrum of manifestations. Besides the Ig composition of cryoprecipitates, their definitive evaluation requires: i. histopathological assessment of microvascular skin lesions, typically non-inflammatory thrombotic lesions in MoCs and leukocytoclastic vasculitis in MCs, ii. detection of immunoserological abnormalities (e.g., complement consumption and circulating autoantibodies), and iii. evaluation of associated hematologic/lymphoproliferative disorders (predominant in MoCs), infectious, and/or systemic autoimmune diseases (more common in MCs).

Finally, a comprehensive multidisciplinary evaluation of the individual cryoglobulinemic patient is essential to determine the most appropriate therapeutic strategy, based on the distinctive etiopathogenetic mechanisms and prevalent clinical features of two major cryoglobulinemia sub-settings.

## Data Availability

The original contributions presented in the study are included in the article/supplementary material. Further inquiries can be directed to the corresponding author.
